# Improved U-Net3+ with stage residual for brain tumor segmentation

**DOI:** 10.1186/s12880-022-00738-0

**Published:** 2022-01-27

**Authors:** Chuanbo Qin, Yujie Wu, Wenbin Liao, Junying Zeng, Shufen Liang, Xiaozhi Zhang

**Affiliations:** 1 Faculty of Intelligent Manufacturing, Wuyi University, Jiangmen, 529020 China; 2 School of Computer and Software, Shenzhen University, Shenzhen, 518000 China; 3 School of Electrical Engineering, University of South China, Hengyang, 421001 China

**Keywords:** Brain tumor segmentation, Stage Residual, U-Net3+, Full-scale connection, FRN

## Abstract

**Background:**

For the encoding part of U-Net3+,the ability of brain tumor feature extraction is insufficient, as a result, the features can not be fused well during up-sampling, and the accuracy of segmentation will reduce.

**Methods:**

In this study, we put forward an improved U-Net3+ segmentation network based on stage residual. In the encoder part, the encoder based on the stage residual structure is used to solve the vanishing gradient problem caused by the increasing in network depth, and enhances the feature extraction ability of the encoder which is instrumental in full feature fusion when up-sampling in the network. What’s more, we replaced batch normalization (BN) layer with filter response normalization (FRN) layer to eliminate batch size impact on the network. Based on the improved U-Net3+ two-dimensional (2D) model with stage residual, IResUnet3+ three-dimensional (3D) model is constructed. We propose appropriate methods to deal with 3D data, which achieve accurate segmentation of the 3D network.

**Results:**

The experimental results showed that: the sensitivity of WT, TC, and ET increased by 1.34%, 4.6%, and 8.44%, respectively. And the Dice coefficients of ET and WT were further increased by 3.43% and 1.03%, respectively. To facilitate further research, source code can be found at: https://github.com/YuOnlyLookOne/IResUnet3Plus.

**Conclusion:**

The improved network has a significant improvement in the segmentation task of the brain tumor BraTS2018 dataset, compared with the classical networks u-net, v-net, resunet and u-net3+, the proposed network has smaller parameters and significantly improved accuracy.

## Background

The precise segmentation of brain tumor regions is an essential basis for clinicians to formulate surgical plans, radiotherapy plans, and pathological examinations. The research of automatic and accurate brain tumor segmentation based on deep learning has made important progress [1, 2]. The improved model based on a fully convolutional network (FCN) [3] and U-Net [4] benchmark network is one of the important research directions. Those improved networks usually have encoder-decoder structure and skip connection structure.

In the multimodal brain tumor segmentation challenge held by Medical Image Computing and Computer-Assisted Intervention Society (MICCAI), most of the participants used U-Net as the benchmark model for model improvement [5–7]. Jiang et al. [5] added a VAE-based image reconstruction branch to the U-Net benchmark network, i.e., a variable auto-encoding branch was added to the decoder structure as a conditional constraint for segmentation. For the limited dataset, it can play a guiding and regularizing effect on the encoder and won the first place on BraTS18. Zhou et al. [6] improved the decoder part and skip connection based on U-Net. They designed architecture with nested and dense skip connections and spliced four U-Net networks of different depths together through multiple skip connections. These skip connections include short and long connections. Short connections enable the gradient to propagate back from the deep decoder to the lower decoder. The long connection is retained because it connects the original feature map of the same scale. The intermediate aggregation feature maps and final aggregation feature maps are connected to restore the information loss caused by down-sampling. The up-sampling uses transposed convolution and finally optimizes the sum of the losses of the four output layers.

Huang et al. [7] believed that U-Net++ was not enough to obtain information from multiple scales. They proposed a new network U-Net3+ (or U-Net+++). U-Net3+ uses full-scale skip connections to combine high-level and low-level semantics from different scales to improve segmentation accuracy, and its network parameters are less than U-Net and U-Net++. The research showed that the encoder part of U-Net3+ is the same as the original U-Net and U-Net++. Each encoder consists of two convolutional layers with a convolution kernel size of 3. Those basic networks can further improve performance. The encoder part can extract the abstract features of the input image. The abstraction degree of features is various under different scales. Higher abstraction degree of features is crutial to the following network. For up-sampling, when the semantic information of the encoder part is combined by skip connection. If the encoder part can provide more semantic information, the segmentation accuracy can be significantly improved.

Gal et al. [8] improved the encoder part based on U-Net combined with the residual structure (Residual Block). Zhang et al. [9] used the residual structure for encoder and decoder. Simon et al. [10] used Dense Block for reference to improve the network structure. The experiments of the above-mentioned improved model showed that the original encoder structure does not absolutely extract features. By combining residual or dense connection structures, the ability to extract semantic information during down-sampling can be improved.

Compared with the two-dimensional (2D) medical image segmentation method, the three-dimensional (3D) segmentation model can make fully use of the 3D sequence-structure information of medical images to fulfill the 3D segmentation task of the lesion [11, 12].

Recently, 3D brain tumor segmentation based on deep learning has performed well [12]. However, the 3D segmentation model still has problems, such as high-computational cost and slow inference process speed. For instance, the 3D V-Net parameters has reached 40 M.

Because of the insufficient feature extraction ability of the encoder part based on U-Net3+, which results in inadequate feature fusion during network up-sampling, reducing the segmentation accuracy, this study proposes an improved U-Net3+ segmentation network based on stage residual and presents its 2D and 3D segmentation models.

First, this study proposes an improved U-Net3+ segmentation network based on stage residual called IResUnet3+. It uses FRN instead of BN to normalize the data after convolution operation; thereby, eliminating the impact of batch size.

Second, from the perspective of the lightweight of the segmentation model, this study develops the IResUnet3+ 3D model from a 3D perspective based on the proposed IResUnet3+ 2D model. At the cost of a smaller number of parameters, it achieves a better segmentation effect than the 2D model. Compared with the 3DV-Net model with 40 M parameters, the same segmentation effect can be achieved.

## Methods

### The dataset

The dataset used in this study is BraTs2018. There are 285 and 66 cases for training and validation set, respectively.

### Proposed model architecture

**Fig. 1:**
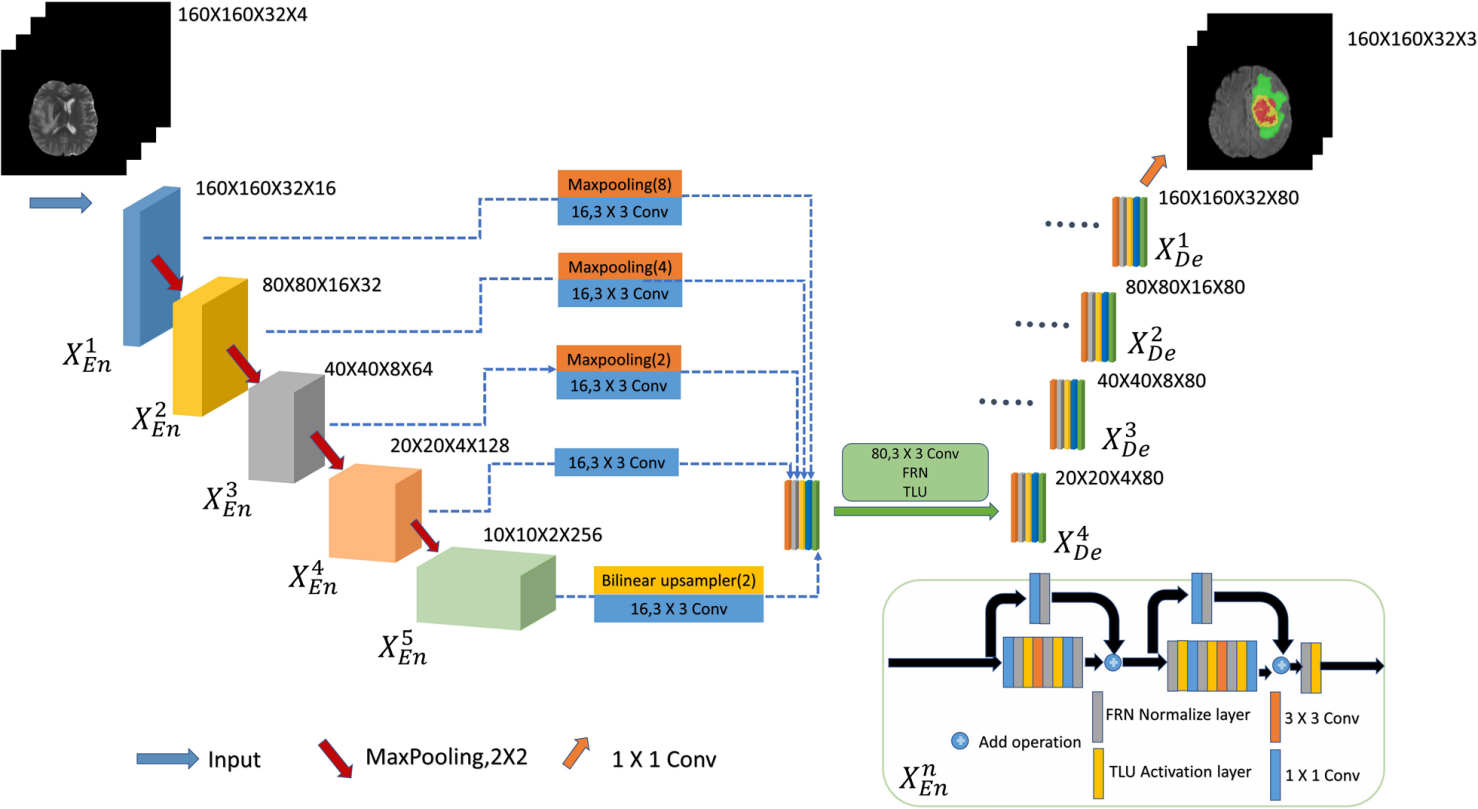
Improved U-Net3+ with stage residual (IResUnet3+) 2D model

This study develops 2D and 3D segmentation models of an improved U-Net3+ segmentation architecture based on stage residual, as shown in Figs. 1 and 6, respectively. The main contributions of this paper are as follows:

1. In the encoder part, an encoder based on the stage residual structure is proposed. This structure reduces the vanishing gradient problem caused by the increase in network depth. Besides, it improves the feature extraction ability of U-Net3+ during down-sampling and provides abundant semantic information for up-sampling.
2. The normalization layer is replaced with FRN [13] instead of BN [14], eliminating the impact on the batch size. The performance can surpass BN when the batch size is large. The network uses an improved version of the ReLU activation function, TLU, which can have certain learning capabilities.
3. Based on the stage residual structure Unet3+ 2D model, we reconstructed the IResUnet3+ 3D model and used block processing to process the 3D data to achieve the 3D network segmentation. The proposed model achieves a segmentation effect similar to the 3D V-Net model with 40 M parameters at the cost of extremely small parameters.

The experimental results showed that the proposed network model improves the segmentation accuracy of small areas, and the edge segmentation of tumors is more smooth and accurate.

### Data preprocessing

**Fig. 2:**
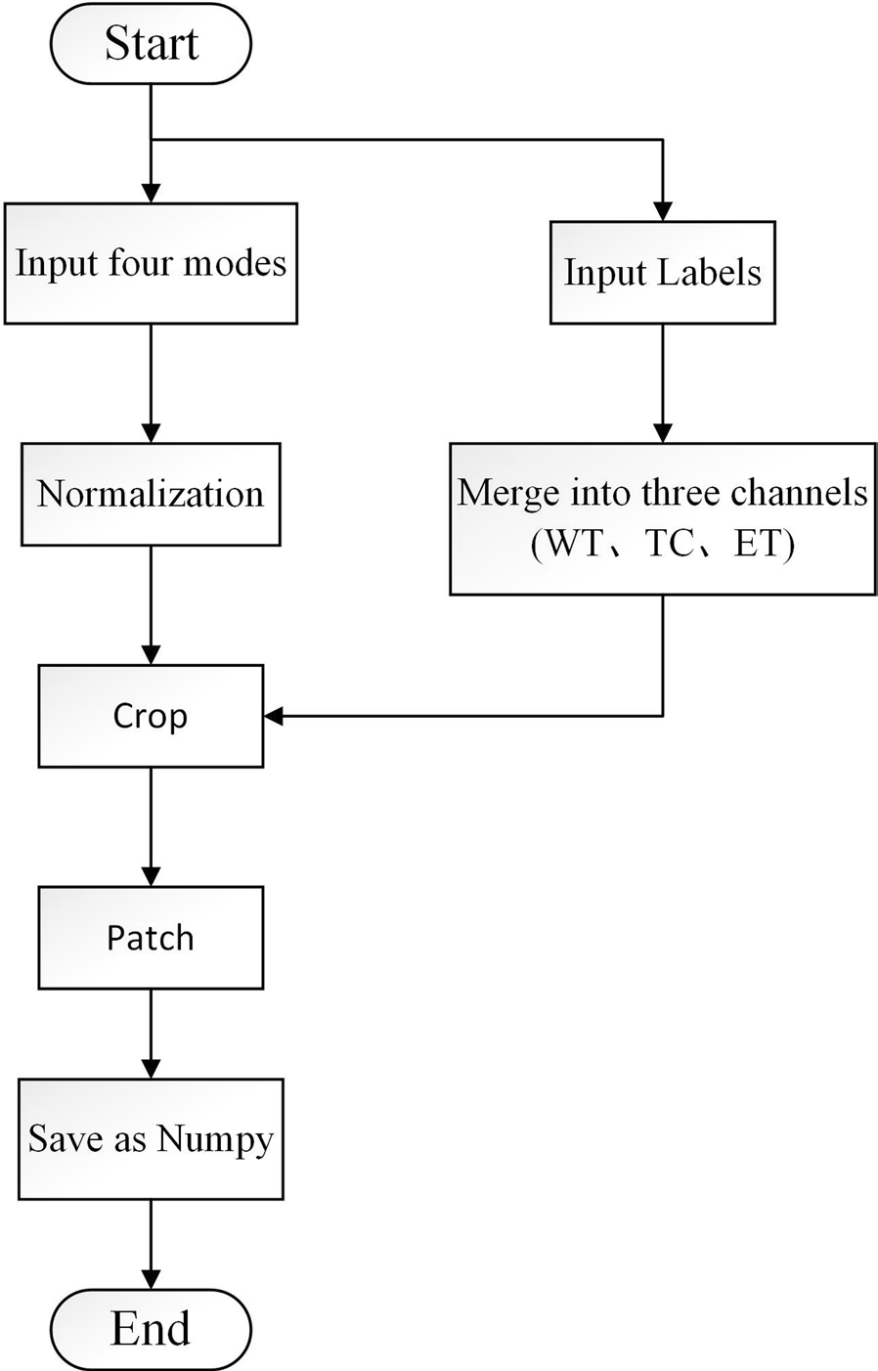
Flow chart of preprocess program

Figure 2 illustrates our data preprocessing. The BraTS2018 contains four modalities as follows: T1-weighted images, T2-weighted images, fluid-attenuated inversion recovery (FLAIR), and contrast-enhanced T1-weighted images (T1C). Since there is contrast difference between each modes, and we use standardization to solve this problem. The corresponding ground truth has three labels: edema area (ED), enhanced tumor area (ET), and non-enhancing tumor (NET). The above labels are divided into three different segmentation nested regions: whole tumor (WT), tumor core (TC), enhancing tumor (ET). Then, merge the channels of the four modalities and the three-segmented regions. After cutting out the redundant background, a making patch is performed to adapt to the 2D network segmentation. Finally, save it as.npy file.

### Encoder based on stage residual

To solve the network vanishing gradient problem, researchers often use the residual structure proposed by He et al. [15] to train deep networks. However, this structure causes some other problems, for example, the number of ReLU on the main path of the residual structure is proportional to the network’s depth. But the information flow with negative weight will be cleared after the ReLU activation function. This feature makes the information flow much affected in the propagation process. To solve such problems, He et al. [16] proposed the pre-activation structure. The principle is to put the ReLU away from the main path. Although the above problem is solved, it causes new problems. Due to the non-linear nature of the activation function, the network can’t learn the non-linear relationship in the data. If there is no non-linear activation function in the residual structure, it will result in the lack of nonlinearity between different residual blocks, which also increases the difficulty of learning the network. The main path of the standard residual and pre-activated structures is not normalized. Thus, the entire signal (the added signal) is not completely normalized, increasing the difficulty of network convergence.

**Fig. 3:**
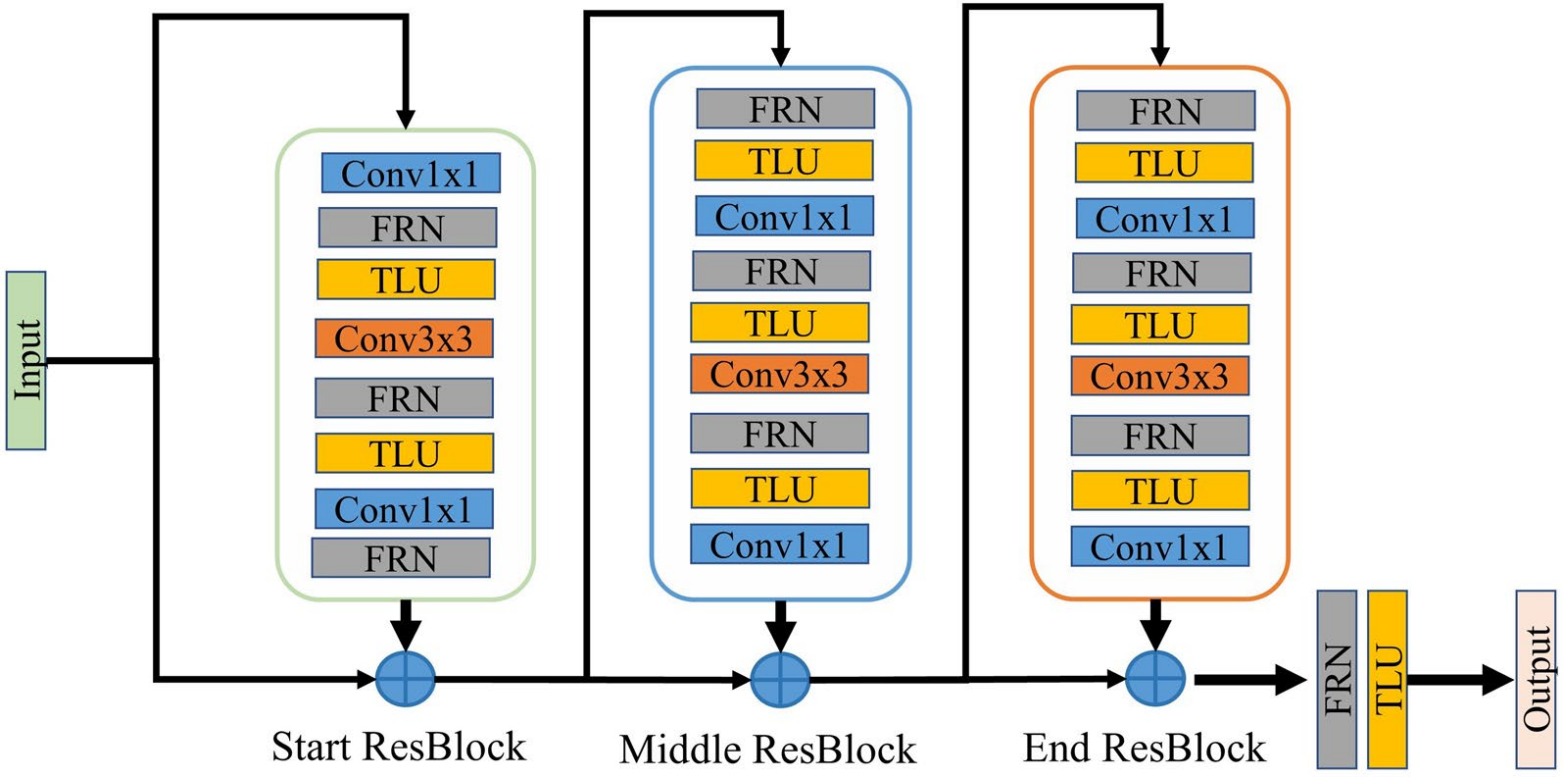
Stage residual structure

Based on this result, Ionut et al. [17] proposed a stage residual structure. As shown in Fig. 3, the principle is to divide the network into different stages. Each stage consists of a start residual block, several middle residual blocks (any number can be used), and an end residual block. Thus, no matter how the network depth changes, if the number of stages remains the same, the number of ReLU on the main path will not change. This allows the signal to reduce many bad effects caused by ReLU when passing through the multi-layer network. It also obtains the non-linear benefits of ReLU. After the end of the residual block, the entire signal is normalized, accelerating the network convergence.

Based on the structural advantages of the stage residual, this study combines it with the encoder to improve the feature extraction ability during down-sampling. The improved encoder consists of a start residual block, several middle residual blocks, and an end residual block. The number of middle residual blocks is set to 0 to ensure that the number of 3 × 3 convolutions is consistent with that in the benchmark network.

### Full-scale skip connection

In addition to improving the encoder part, skip connections are the focus of attention, such as U-Net++ [6] designed architecture with nested and dense skip connections based on U-Net. However, Huang et al. [7] believe that U-Net++ does not have enough information from multiple scales; thus, they proposed U-Net3+. It uses full-scale skip connections to combine high-level and low-level semantics from different scales to provide richer information for up-sampling.

**Fig. 4:**
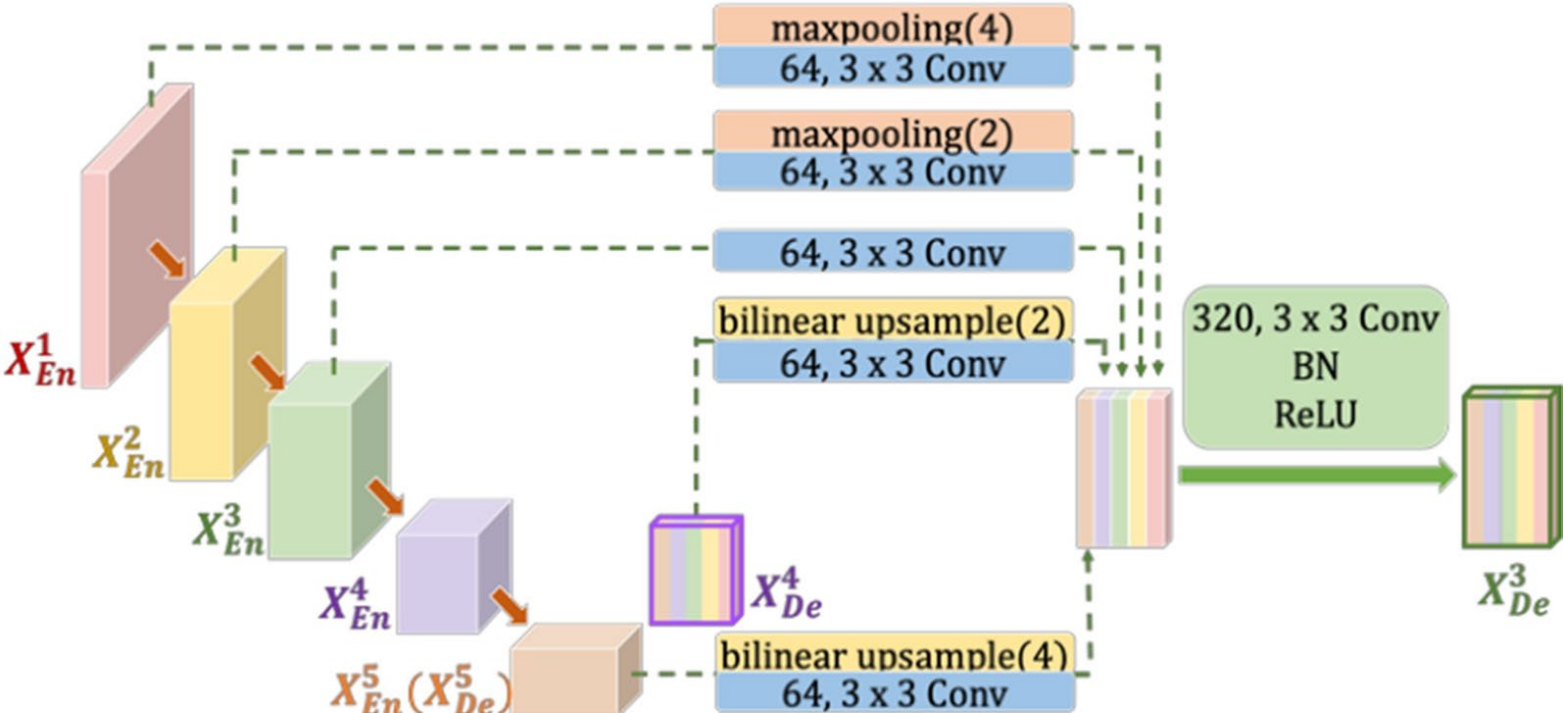
How to construct decoder with Full-Scale skip connection (as an example)

Figure 4 explains how to structure the ${X}_{De}^{3}$ feature map. Similar to U-Net, directly receive feature maps ${X}_{En}^{3}$ from the same scale encoder layer. But the difference is that there is more than one skip connection above. Among them, the above two skip connections perform pooling down-sampling of the lower-level encoder layers ${X}_{En}^{1}$ and ${X}_{En}^{2}$ through different max pooling operations to transmit the low-level semantic information. Another reason for pooling down-sampling is to unify the resolution of the feature map. It can be seen from the Fig. 4, ${X}_{En}^{1}$ has to reduce the resolution four times and ${X}_{En}^{2}$ has to reduce the resolution two times. The next two skip connections use bilinear interpolation to up-sample ${X}_{En}^{4}$ and ${X}_{En}^{5}$ in the decoder to enlarge the resolution of the feature map. It can be seen from the figure, ${X}_{En}^{5}$(${X}_{De}^{5}$) has to enlarge the resolution four times and ${X}_{En}^{4}$ has to enlarge the resolution two times. After unifying the size of the feature maps, it is necessary to unify the number of channels. After convolution through 3 × 3 convolution with 64 channels, it will be concatenated together along the channel dimension, and then the feature fusion is performed. After fusion, a new feature map with 320 channels is generated. Finally, ${X}_{De}^{3}$ can be obtained by Conv-BN-ReLU.

### FRN

The experimental comparison showed that in each stage, no matter U-Net, U-NET++, or U-Net3+, batch normalize is used to normalize the data passing through the convolution layer, which makes the whole network limited by the batch size N [14]. When the batch size N is small, the network effect will be very poor. Although group normalization proposed by He is not affected by the batch size, it has not been widely used [18]. Besides, it is not easy to compete with BN when the batch size is large. FRN breaks the influence of batch size and surpasses BN when batch size is large [13].

**Fig. 5:**
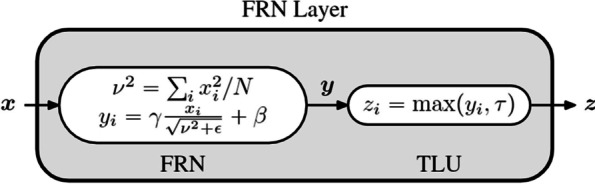
Calculation process of FRN (N is the batch size, C is channel number, H, W is characteristic graph size)

Figure 5 illustrates the calculation process of FRN. The input data X refers to the data of a characteristic graph (H, W); thus, it has nothing to do with the N representing the batch size. Its calculation process is slightly different from other normalization layers [14, 21]. It omits the operation of subtracting the mean value and changes the variance to the mean value of the quadratic norm of ${v}^{2}$. Similarly, scaling and panning are required after normalization. Where $\in$ is a small constant to prevent the denominator from being zero. Besides, FRN does not perform any subtraction of the average value, so it may lead to the result far from zero after normalization. When FRN is activated by ReLU after normalization, many 0 or 1 values may be generated, which is detrimental to model training and performance. To solve this problem, we use threshold ReLU to eliminate the bias phenomenon, namely TLU, as shown below:

1 $$Z = \max (y,\tau ) = {\text{Relu}}(y - \tau ) + \tau$$

The parameter $\tau$ is learnable. Saurabh et al. [13] found that TLU is very important to improve the performance after FRN normalization.

### Loss function

In medical image segmentation, data imbalance is a very common problem. In general, the number of lesion voxels in most datasets is much lower than that of non-lesion voxels, the same is true for brain tumor datasets, and the area of brain tumor is much smaller than that of the brain region. To solve this problem, Fausto et al. [11] proposed a loss function based on the Dice coefficient, which significantly alleviates this imbalance phenomenon and makes the network learn effectively. But for small target segmentation, once it is not detected, Dice loss fluctuates violently. Thus, this study selects the mixed loss function of the combination of cross-entropy loss and generalized Dice loss and gives them corresponding weights. The formula is shown below.

2 $$L_{{{\text{all}}}} = \alpha L_{bce} + \beta L_{dice}$$

The parameters of loss function are α = 0.5 and β = 1.0.

### 3D model based on IResUnet3+

**Fig. 6:**
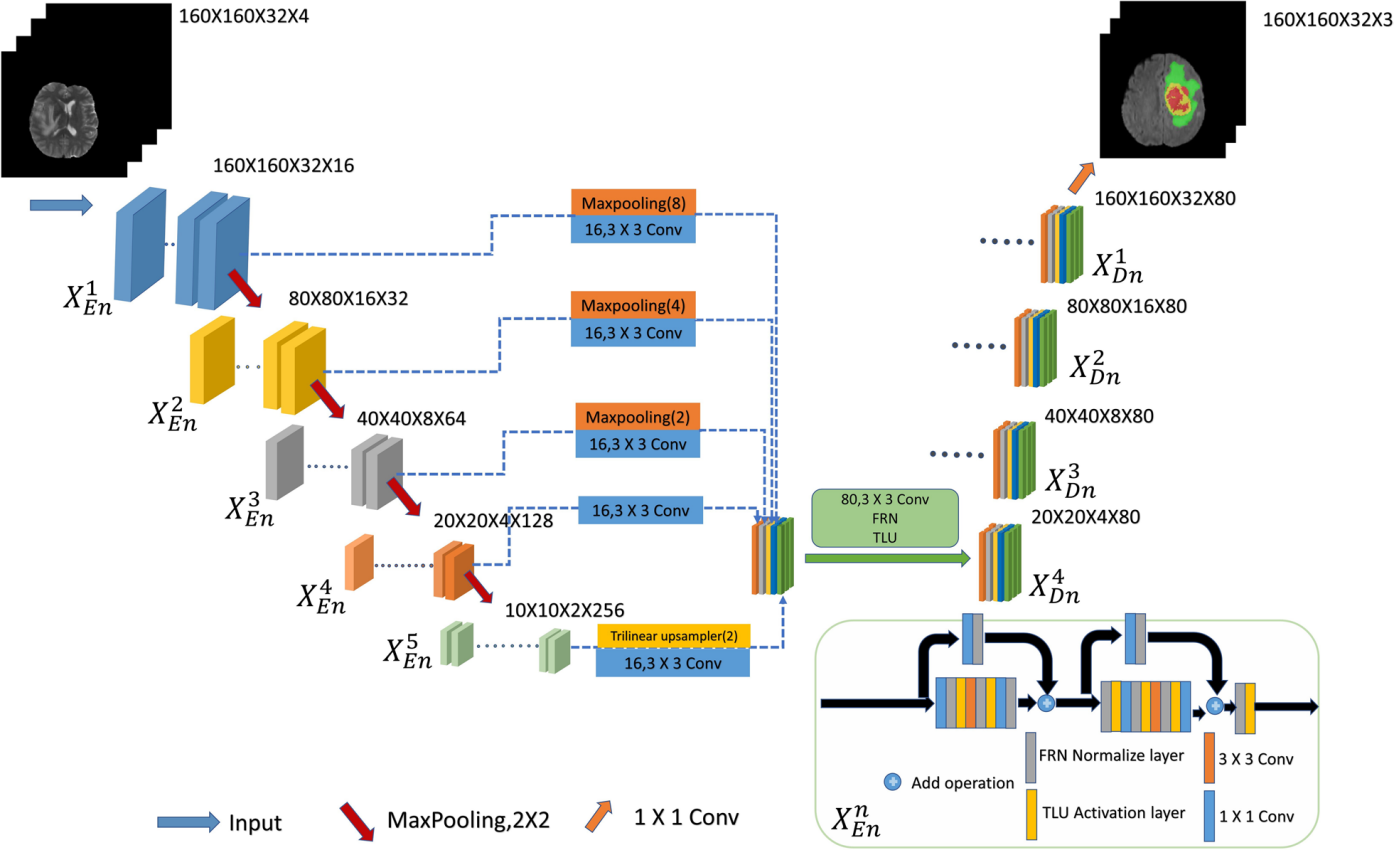
Improved U-Net3+ with stage residual (IResUnet3+) 3D model

In the front part, a 2D neural network is used to segment brain tumor magnetic resonance imaging (MRI). Although the proposed IResUnet3+ network has been significantly improved, there are still some false alarms in the normal tissue areas around the brain tumor, i.e., many outliers are predicted in the surrounding areas. This is because the MRI sequence is originally 3D data, but it is sliced in the preprocessing of the 2D network, which causes the patch data to lose much spatial information, leading to insufficient network learning. Thus, this study develops the IResUnet3+ 3D model to discuss the effect of 3D model brain tumor segmentation based on the proposed IResUnet3+ 2D model. The structure of the proposed IResUnet3+ 3D model is the same as that of the 2D model, except that 3D convolution is used instead of 2D convolution, and FRN and TLU are improved to adapt to the 3D input data. The major difference from the 2D model is the data preprocessing part; it will be explained in detail in subsection 3.1. The IResUnet3+ 3D model diagram is shown in Fig. 6 below.

### 3D data preprocessing

**Fig. 7:**
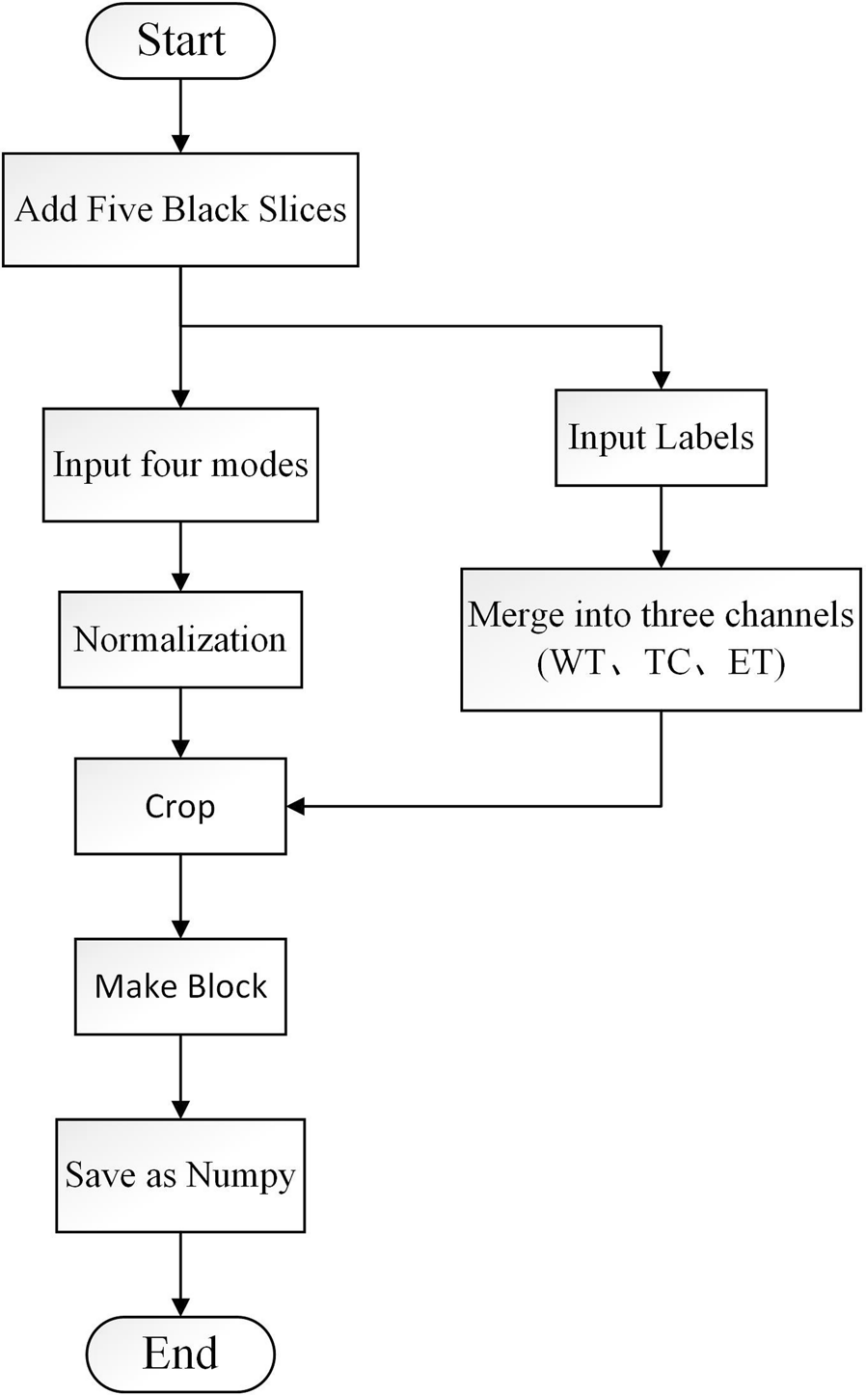
Flow chart of 3D data preprocess program

**Fig. 8:**
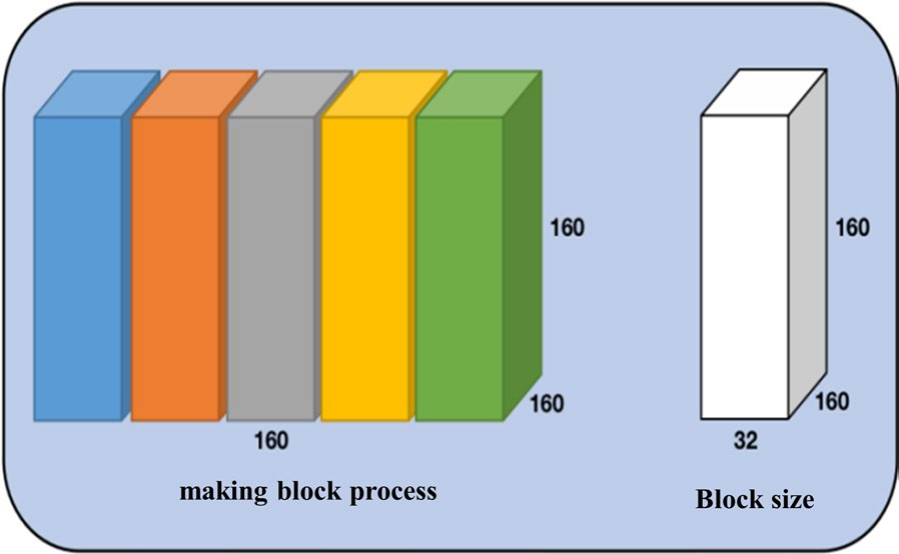
Method A to divide into blocks

Due to the limited experimental resources and conditions, it is not possible to directly input the complete 3D data into the network. To achieve the 3D network segmentation, the 3D data is divided into blocks. Different from the making patch of the 2D network, the block data is still 3D data. As shown in the Fig. 7, The preprocess method is divided into five steps: First, manually add five black slices to meet the requirements of the block method. Add three black slices to the front of the four modal images (155, 240, 240) and the corresponding mask (155, 240, 240). Then, add two to the back, and finally, all become (160, 240, 240). After normalization and crop, block processing is conducted. Figure 8 shows one of the making block methods A, the cropped image and label size are both (160,160,160), the block size set is (32,160,160), the moving step is 32, this means five blocks of (32,160,160) size are divided from the Z-axis direction. Therefore, the data size input to the network is (BS, 4, 160, 160, 32). And 4 are images of four modes.

**Fig. 9:**
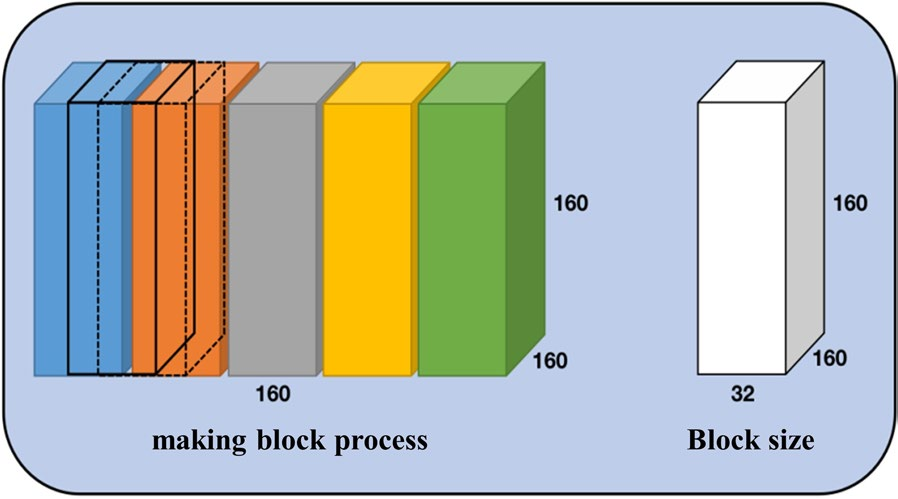
Method B to divide into blocks

Data preprocessing plays a decisive role in model training. Poor preprocessing may result in insufficient training or even failure. For the data preprocessing of 3D networks, the making block method is a step worth paying attention to. After experiments, although the above making block method (Fig. 8) is simple, there is a lack of correlation between blocks, making the network unable to fully learn the structural relationship between all blocks and blocks while training. The block at this moment is similar to the slice in the 2D network. Although it contains more 3D structural information than the slice, there is still a lack of connection between the blocks. Besides, the network cannot learn the interconnection between structures. Thus, we explored another making block method, as shown in Fig. 9, and called it making block method B for distinction. Making block method B is also simple. The size of the block is not changed, but the moving step of the block is set to 8, i.e., a block of (32,160,160) size is taken for every eight movements in the Z-axis direction.

**Fig. 10:**
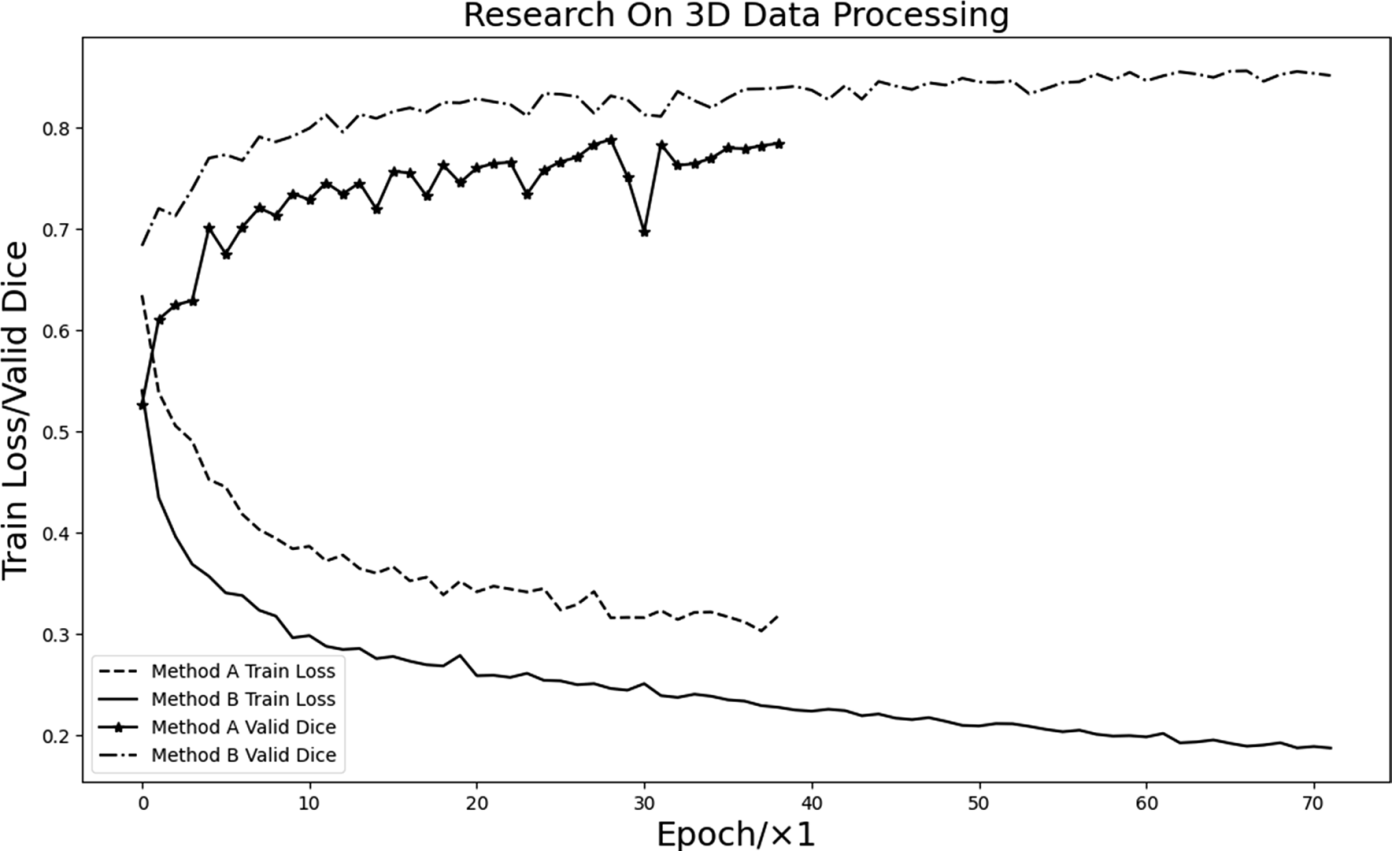
Block data A and B in V-Net network training process

To compare the differences between the two making block methods, method A and method B are used to process the data, and the V-Net network is trained under the same experimental conditions. At the same time, set the early stopping method to supervise the training process. When the accuracy of the validation set does not improve after a certain number of epochs, the early stopping method is triggered to end the training. Figure 10 shows the comparison of the training process on the data obtained in the two methods. The Fig. 10 shows that the data obtained in method A lacks the mutual connection information between blocks so that the model cannot be fully trained. When the early stopping method is triggered, the model loss remains at a high level. The data obtained in method B enables the network to fully learn the 3Dl structural information of all data in the dataset, which is beneficial to the convergence and accuracy of the network.

In summary, data preprocessing plays a pivotal role in model training. Compared to making block method A, making block method B allows the data in the dataset to be related to each other, allowing more structural information to be captured during model training, which is conducive to network learning and training.

## Experiment and analysis

### Experimental environment

The dataset used in this study is BraTS2018. There are 285 and 66 cases for training and validation set, respectively. The operating environment: Win10, Intel Core i7-8700@ 3.20 GHz six-core CPU, memory 32 GB, graphics card Nvidia GeForce GTX 1080Ti (11 GB/Gigabyte), Pytorch1.4.0, Python3.6. The Adam optimizer is used for gradient descent, the learning rate is 0.03, and the batch size is 2.

### Analysis of feature extraction ability

**Fig. 11:**
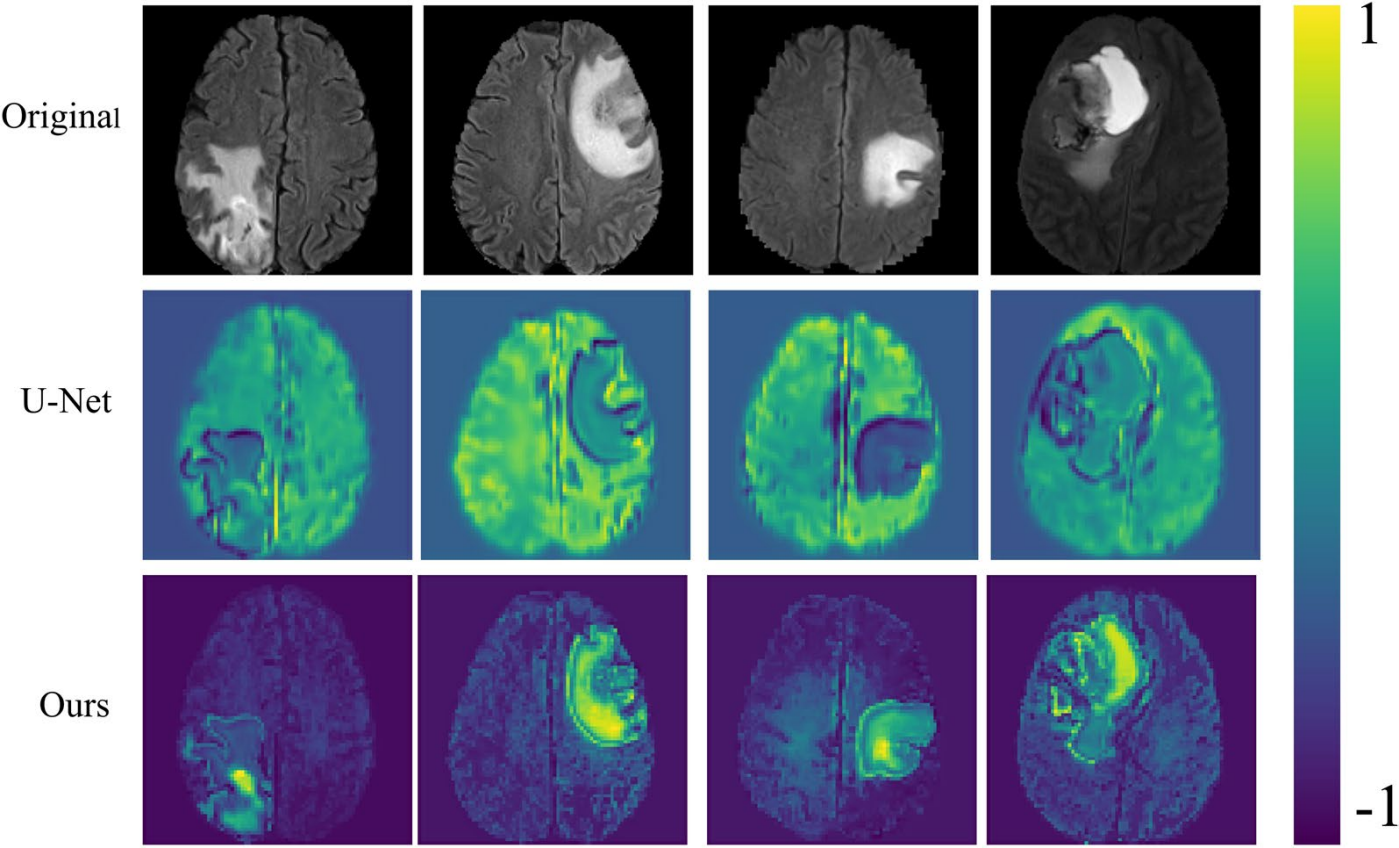
Feature extraction visualization

In medical image segmentation, we expect to obtain a binary image that only contains the lesion location (a lesion location is positive number, and the rest is 0). Therefore, our neural network model should have the ability to identify the lesion location, highlight the lesion location, and weaken the non-lesion location. And the feature extraction ability of the model is also reflected in the perception of the lesion location. In 2.2, we mentioned that the feature extraction ability of the improved encoder based on stage residual has been significantly improved. Therefore, we show the output results of the proposed model’s encoder layer through the visual method and compare it with U-Net. As shown in Fig. 11, the U-Net model has a poor perception of the lesion location in the input image, and the model's attention is scattered throughout the image instead of the lesion location. And the proposed model is very sensitive to the lesion location and can better identify and highlight the lesion location and weaken the non-lesion location. This also indicates that the feature extraction ability of the encoder is improved after adding the stage residual structure.

### Experimental comparative analysis

**Fig. 12:**
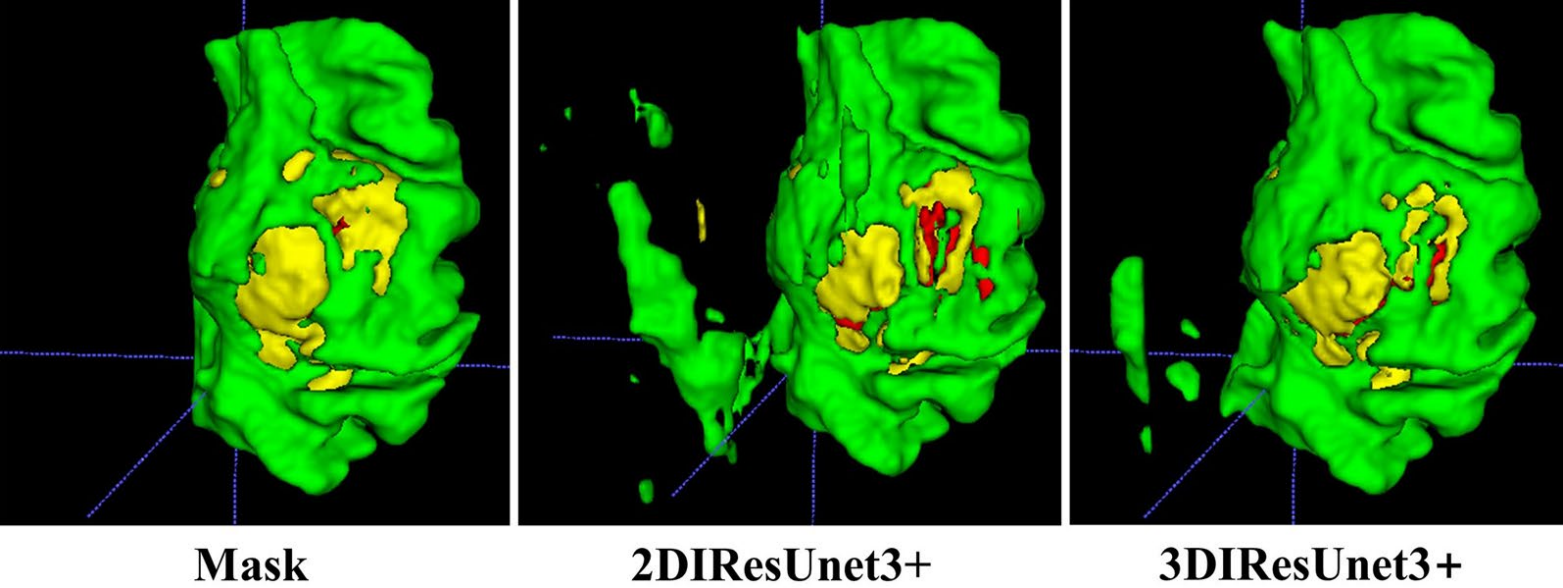
Comparison of segmentation effect between 2 and 3D models

**Fig. 13:**
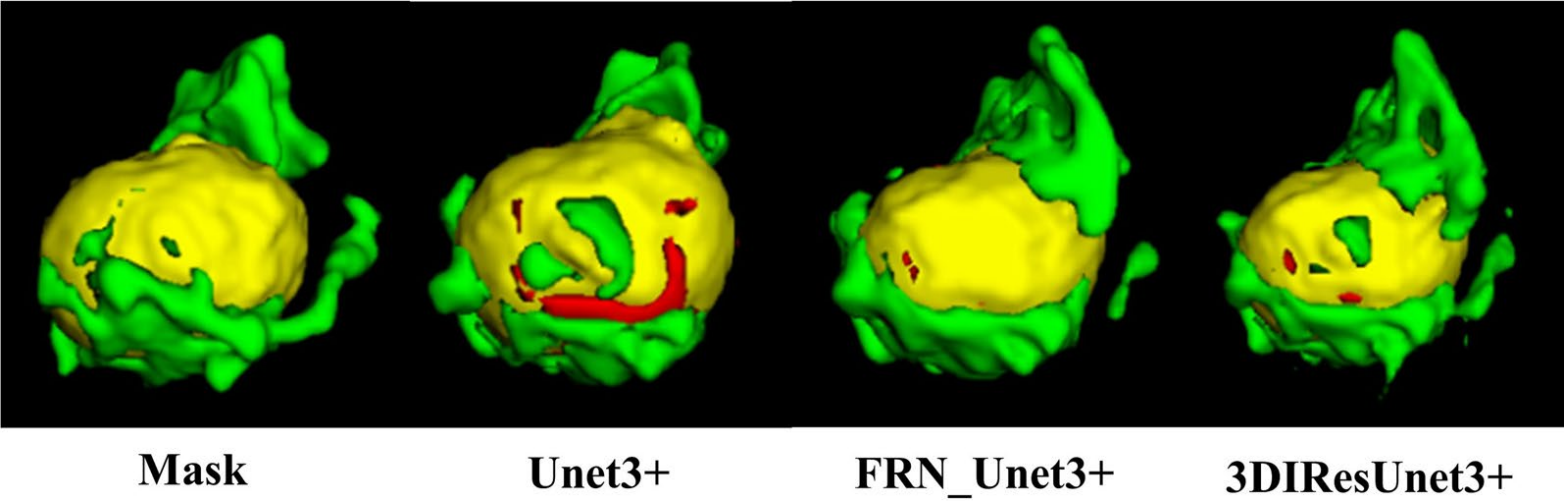
Comparison of U-Net, U-Net++, and U-Net3+ segmentation results

**Fig. 14:**
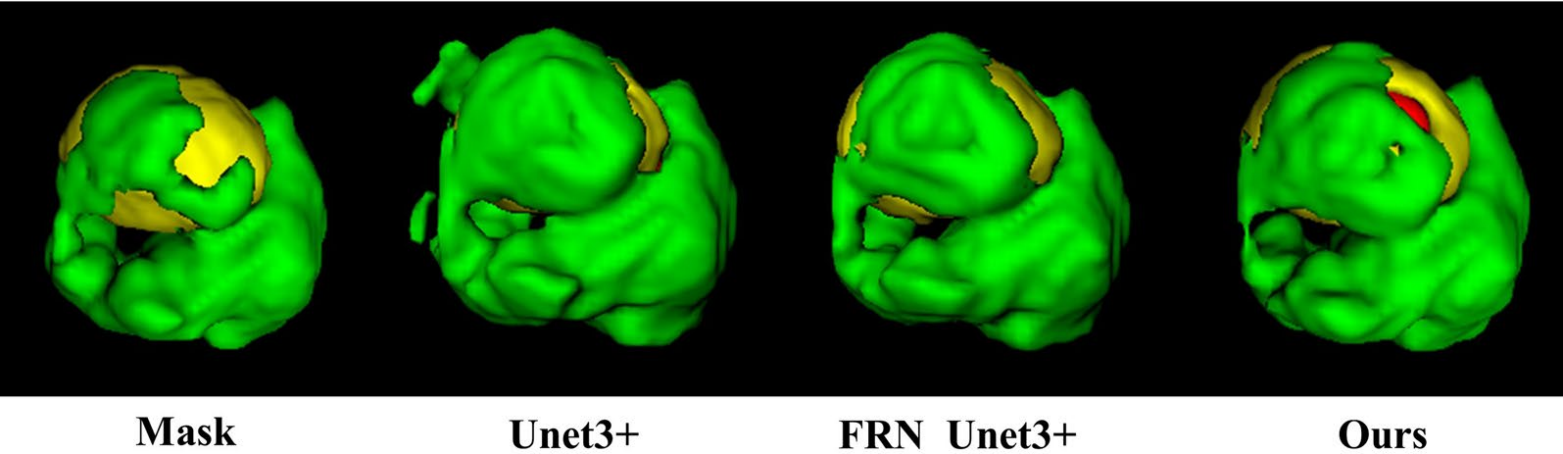
Comparison of ablation experiment

On the same dataset, the proposed model is tested and compared with the existing mainstream models. 2D and 3D models are constructed to examine the difference in the performance of the 2D and 3D models under the task of brain tumor segmentation. The mainstream medical image segmentation models used for comparison are U-Net, U-Net++, U-Net3+, and ResUnet, with the experimental results shown in Figs. 12, 13, and 14, respectively. Among them, green area represents peritumoral edema area(ED), yellow represents enhancing tumor area (ET), and red represents non-enhancing tumor(NET).

### Comparative analysis of 2D and 3D models

Comparing the 2D and 3D models of IResUnet3+ on the segmentation effect of brain tumors, the 2D model has a large area of misjudgment and additional judgments when predicting the 3D brain tumor data. This is because the input data of the 2D model is one picture by one. The network cannot learn the connection between pictures. The input data of the 3D network is a 3D block, which itself contains 3D structural information. Besides, we use making block method B. The network can further obtain the connection between blocks, and further help the network to learn the 3D structural information of tumor lesions, improving the accuracy of brain tumor segmentation and reducing misjudgment rate.

### Ablation study

Comparing the segmentation effect of U-Net, U-Net++, and U-Net3+ models for brain tumors, we obtained that the classical network U-Net is based on its encoder-decoder network structure, and skip connection can connect encoder-decoder layer to merge low-level and high-level features to better perform basic segmentation of tumor lesions. However, there are still many problems, such as misjudgments, additional judgments, and low accuracy. U-Net++ designs architecture with nested and dense skip connections based on U-Net. The four U-Net networks of different depths are spliced together through multiple skip connections, which help to fully integrate features at the same scale. However, it does not perform feature fusion between different scales, and there may be a problem with feature redundancy. Based on this, U-Net3+ is proposed. U-Net3+ proposes a full-scale skip connection while retaining the simple architecture of U-Net one-layer encoder-decoder. The features from different scales are merged through skip connection without feature redundancy. All feature information of different scales appears and is integrated. In contrast, U-Net3+ can achieve better results on segmentation tasks.

Comparing the segmentation effects of U-Net3+, FRN_U-Net3+, and IResUnet3+ models on brain tumors, we obtained that, as described in the previous section, U-Net3+ can segment brain tumors due to its full-scale skip connection. However, it needs further improvements. First, the BN normalization method used in U-Net3+ will limit the network to the batch size. When the batch size is small, the network performance tends to be poor. Thus, we used the FRN normalization layer instead of BN to eliminate the batch size impact on the network. under the same batch size training, FRN_U-Net3+ performs significantly better than U-Net3+. It is essential to eliminate the influence of batch size on the network. The traditional Conv-BN-ReLU operation is used in the U-Net3+ encoder part, which shows weak feature extraction ability. We used the improved encoder based on the stage residual to improve the feature extraction ability of the encoder part, which is helpful for the network to learn more feature information and conducive to better feature fusion in the up-sampling.

**Fig. 15:**
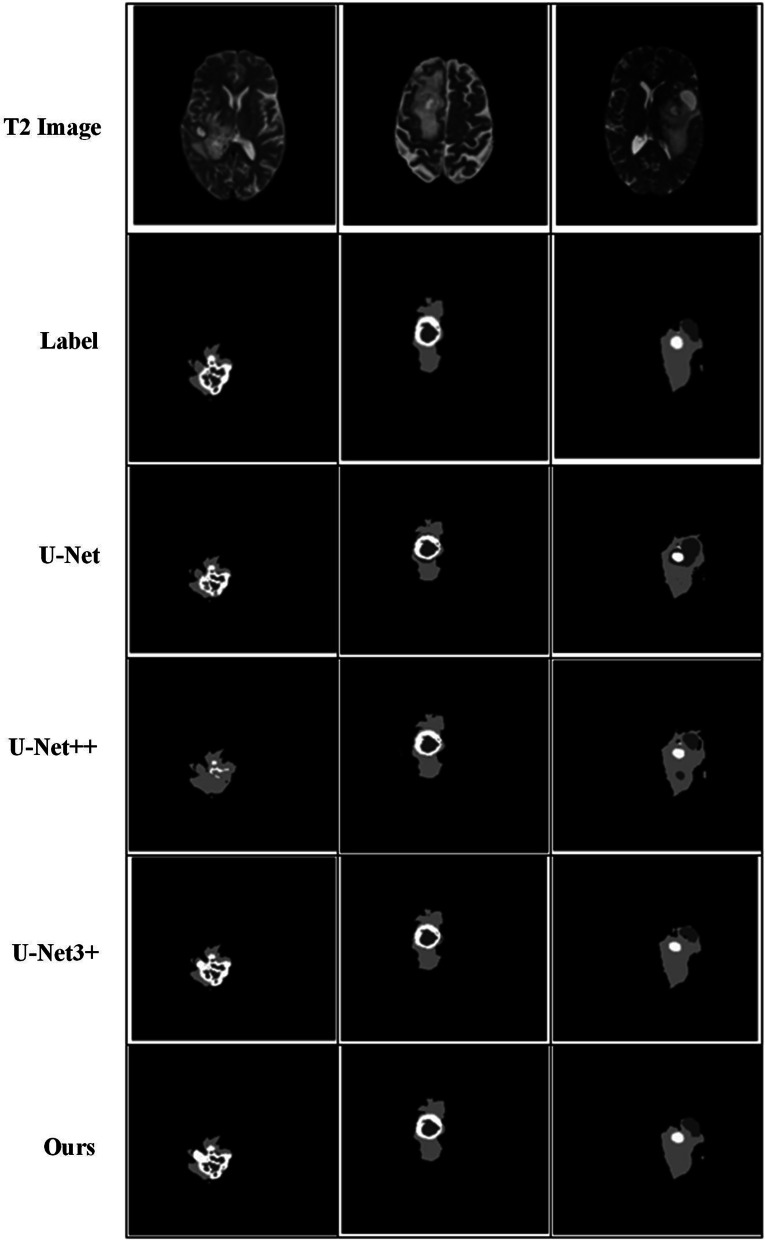
Segmentation result of all 2D models

**Fig. 16:**
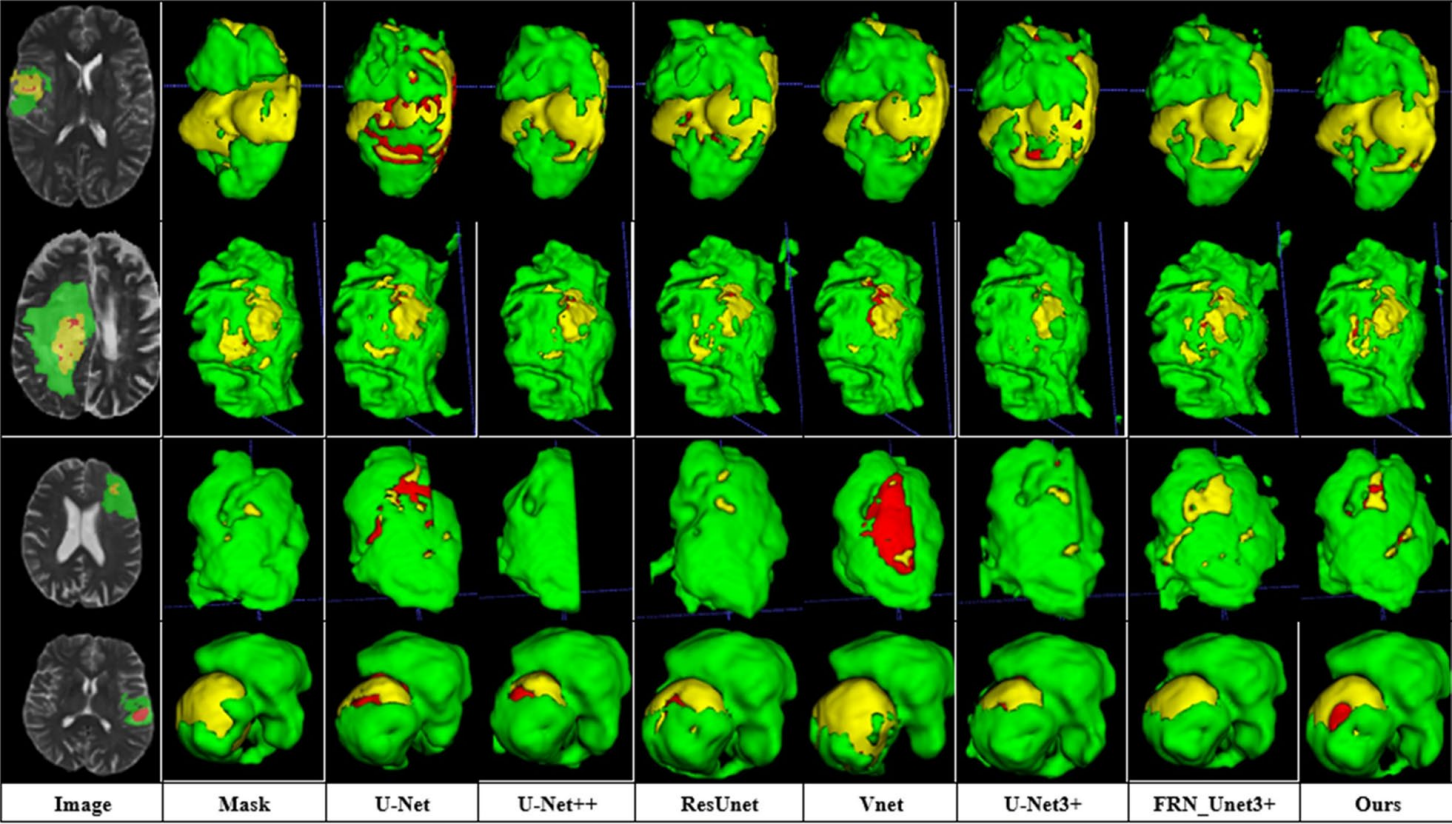
Segmentation result of all 3D models

Finally, all 2D and 3D model segmentation results are shown in Figs. 15 and 16, respectively.

### Statistical analysis of segmentation results

**Fig. 17:**
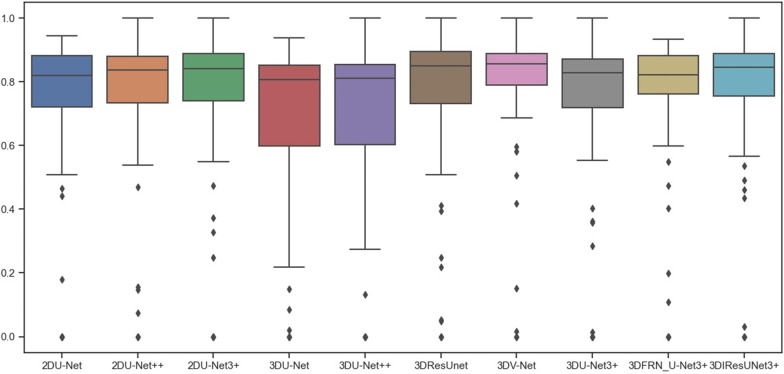
Box plot of all experimental models

We evaluated all models using the validation dataset provided by the BraTS2018 challenge. segmentation result of each architecture is evaluated as presented in Table 1. Box plots of all experimental models are displayed in Fig. 17. And the standard deviation and confidence interval of the scores of our proposed model are shown in Table 2. Note that all metrics are calculated through the BraTS2018 online evaluation platform. And two commonly used medical image segmentation evaluation indexes are used to evaluate the segmentation results: Dice coefficient (Dice), Sensitivity (SEN). Moreover, each index has segmentation results corresponding to three regions: whole tumor (WT), tumor core (TC), enhancing tumor (ET).The formula is shown in (5). Among them, TP is the number of pixels with correct foreground segmentation in pixel-level segmentation, FP is the number of pixels with background segmentation error in pixel-level segmentation, and FN is the foreground segmentation error in pixel-level segmentation. Among them, Dice is used to calculating the similarity between prediction results and labels, and the greater the similarity of Dice, the higher the similarity. And SEN indicates the probability that the lesion will be correctly segmented

3 $$\begin{aligned} & {\text{Dice}} = \frac{2TP}{{2TP + FN + FP}} \\ & SEN = \frac{TP}{{TP + FN}} \\ \end{aligned}$$

**Table 1:** Segmentation effect of each model

Model Type	Params	ET Dice	WT Dice	TC Dice	SEN_ET	SEN_WT	SEN_TC
2DUnet [4]	39 M	72.34	86.22	73.77	77.80	85.81	71.47
2DUnet++ [6]	36 M	72.39	85.60	73.36	76.20	85.78	71.81
2DUnet3+ [7]	27 M	73.93	87.23	77.28	74.94	88.26	77.97
Swin transformer [19]	27 M	76.18	83.75	**82.33**	78.31	84.28	80.34
3DUnet [20]	4.1 M	67.12	87.37	73.52	65.35	89.01	80.62
3DVnet [11]	40 M	**76.25**	**88.87**	78.72	80.00	91.30	**83.37**
3DResUnet [9]	4.2 M	72.60	87.96	71.24	73.70	90.11	73.43
3DUnet + +	6.8 M	67.12	85.81	67.66	63.91	88.38	75.99
3Dunet3 +	5 M	72.41	86.89	73.53	72.74	90.94	76.60
3D_FRN_Unet3+	5 M	72.22	87.74	78.59	**81.18**	**92.28**	81.20
Ours	6.6 M	75.65	88.77	78.62	79.12	91.51	78.96

Bold indicates that the value is the maximum value in the index

**Table 2:** The standard deviation and confidence interval of our proposed model

	Dice_ET	Dice_WT	Dice_TC	Sen_ET	Sen_WT	Sens_TC
SD	0.24757	0.07148	0.20138	0.22927	0.08815	0.22575
Median	0.84598	0.91022	0.86591	0.85295	0.93605	0.86607
25 quantile	0.75632	0.8663	0.71147	0.73508	0.89561	0.71026
75 quantile	0.88787	0.93284	0.91945	0.94034	0.96758	0.95347

We obtain the following from the table. First, comparing the segmentation effect of 2D and 3D models, we obtain that the 3D segmentation model has some problems, such as high-computational cost and a large amount of memory. Thus, we have to reduce the scale of the model to reduce its parameters (here, the convolution channel number of each layer of the 3D model is [16,32,64,128,256], while that of the 2D model is [64,128,256,512,1024]; thus, reducing the scale of the 3D model). However, it will decrease the learning ability of the model, and the final segmentation effect will be worse. The proposed model can maintain good learning ability and improve the segmentation effect under the same compression model scale. Second, comparing 3DU-Net, 3DU-Net3+, 3D_FRN_Unet3+, and 3DIResUnet3+, we obtain that the full-scale skip connection proposed by U-Net3+ can provide more information for up-sampling by combining high-level and low-level semantics from different scales; thus, improving the segmentation accuracy. Due to the common BN normalization layer used in U-Net3 + , the network is limited by the batch size. Thus, we use the FRN normalization layer instead of BN to solve the problem of the network limited by the batch size, so that the network can be fully trained, and the segmentation accuracy is improved. The Dice coefficients of ET and TC increased by 0.85% and 5.06%, respectively. The sensitivity of WT, TC, and ET increased by 1.34%, 4.6%, and 8.44%, respectively. The encoder is improved based on the stage residuals, which solves the problem of insufficient feature extraction ability of U-Net3+ encoder at the cost of adding a small number of parameters, and provides more semantic information for up-sampling to further improve the segmentation accuracy. The Dice coefficients of ET and WT were further increased by 3.43% and 1.03%, respectively. Moreover, the standard deviation of Dice-ET, Dice-WT and Dice-TC indexes of the model is 0.24757, 0.07148, 0.20138, the median value is 0.84598, 0.91022, 0.86591, and the 75quantile is 0.75632, 0.8663, 0.71147. Comparing 3D IResUet3+ with 3D V-Net, we obtained that the proposed model can achieve the segmentation effect similar to the 3D V-Net model with 40 M parameters with minimal parameters. Thus, the IResUnet3+ model is lightweight and effective in brain tumor segmentation.

The statistical analysis experiment is shown in Fig. 17. Under the same experimental conditions, we have statistically analyzed the ET-Dice index in the experimental results. The analysis methods of other indexes are the same and are not displayed one by one. The models used are: 2DU-Net, 2DU-Net++, 2DU-Net3+, 3DU-Net, 3DU-Net++, 3DResUnet, 3DV-Net, 3DU-Net3+, 3DFRN_U-Net3+ And our proposed model 3DIResUnet3+. By observing the Box diagram, it can be found that with the gradual improvement of 3DUnet3+, we gradually add FRN module and stage residual module to obtain 3DFRN_UNet3+ and 3DIResUnet3+ models, the average value of the Box diagram drawn by them increases gradually, so the generalization accuracy of the model also increases gradually, which is consistent with the experimental data in Table 1.

The HD distance results of all models are shown in the following Table 3. It can be seen from the Table 3 that the Hausdorff distances of ET, WT and TC of 2DUnet are 11.02, 25.71 and 17.65, and our proposed model has improved by 2.7, 12.63 and 2.46 compared with it. And compared with 3DUnet, Table 1 above shows that the Dice values of ET, WT and TC in our proposed model have increased by 8.53, 1.4 and 5.1 respectively. Table 3 shows that HD-ET has increased by 19.96, but the values of HD-WT and HD-TC have decreased slightly. This shows that the index HD sensitive to the difference of location information will be slightly affected while increasing the value of dice.

**Table 3:** Statistical results of Hausdorff distance

Model type	Params	HD-ET	HD-WT	HD-TC
2DUnet [4]	39 M	11.02	25.71	17.65
2DUnet++ [6]	36 M	11.06	32.42	21.96
2DUnet3+ [7]	27 M	6.97	17.54	11.98
3DUnet [20]	4.1 M	28.28	9.03	13.79
3DUnet++	6.8 M	6.01	8.33	12.36
Ours	6.6 M	8.31	13.08	15.19

## Conclusions

Focus on the problem that the encoder of U-Net3+ has insufficient ability to extract features. In this study, an improved encoder structure based on stage residuals is proposed to improve the feature extraction ability in down-sampling. We used the FRN normalization layer to eliminate the impact of batch size on the network. The IResUnet3+ 3D model is constructed based on the stage residual structure Unet3+ 2D model. The 3D data is processed to achieve accurate segmentation of the 3D network. The proposed IResUnet3+ 3D model achieves a segmentation effect similar to that of the 3DV-Net model with 40 M parameters at the cost of minimal parameters, which is lightweight and effective in brain tumor segmentation tasks. The experimental results showed that the improved network could significantly improve the segmentation accuracy of the brain tumor BraTS2018 dataset compared to the original U-Net3+ . The next step is to study the 3D segmentation and localization of brain tumor images and establish a prediction model combined with radiomics to improve the diagnosis, treatment, and prognosis of brain tumors.

## Data Availability

The database used in this paper is BraTs2018, which can be applied from this web page: https://www.med.upenn.edu/sbia/brats2018/data.html.

## Abbreviations

DC: Dice coefficient
SEN: Sensitivity
MRI: Magnetic resonance imaging
CNN: Convolution neural networks
BN: Batch normalization
FRN: Filter response normalization
ET: Enhancing tumor
WT: Whole tumor
